# Hospital Finance

**Published:** 1922-06

**Authors:** 


### HOSPITAL FINANCE.

### A CONCESSION TO PRIVATE HOSPITAL PATIENTS.

THE Committee of King's College Hospital, where about a year ago two wards were reserved for private patients at a maintenance charge of five guineas, is extending this scheme. It hopes to devote a third ward, mainly for cancer, and to relate the scheme to the combined hospital appeal. The annual subscriber of one guinea who, or whose wife, or child under sixteen, needs hospital treatment, can be admitted to these wards on the recommendation of his private medical adviser. The patient will pay his medical fees in the usual way, but if he cannot afford the hospital charges, he can claim to be credited with the amount of his annual susbcriptions since January, 1920, and to that extent reduce his expenses. The plan appears to resemble that of the contributors' certificates at the Royal Northern Hospital, except that it is limited to annual subscribers of one guinea, and applies therefore to the private patient in the private ward. The variation should prove welcome and attractive, incidentally to "Truth," whose pages have lately advocated the use of closed wards for private patients, where possible, in the manner adopted at King's.

### GUARDIANS AND PAYING PATIENTS.

ONE of the signs of the times is the effort or proposal made by various guardians to provide for paying patients The case was well put by Captain Hening at a recent meeting of the Wirral Guardians. He said that two other unions had tried the scheme, and another speaker declared that the Birkenhead Board of Guardians had made a profit by it. The object was to increase the inadequate accommodation in hospitals, and to provide an alternative to expensive nursing homes. The proposed charges were four guineas or three guineas a week. Dr. Yeoman, the Medical Officer, favoured the scheme, which was eventually referred to the Infirmary Committee. The general feeling was that surplus accommodation should be so used, but that to build a hospital for paying patients would be *ultra vires.* All this is a welcome symptom of the desire for co-operation in hospital work, and in it may be seen the germ of an adequate hospital service.

### CO-OPERATIVE BUYING IN IRELAND.

AN interesting but apparently abortive attempt has been made by the Dail Local Government Board to arrange for co-operative buying in the interests of Irish institutions. The proposed system is, briefly, as follows. The Department, having collected information from all public bodies of their needs for, say, three months, will invite tenders, and knowing the amounts required, will deal direct with the manufacturers, which each institution cannot separately do. The department will act as broker, making no charge for its services. It will fix the price for a given quantity for a given time, and leave the manufacturer to deliver the goods and collect the payment. The small institutions will be as favourably placed as the large, and the large will benefit because the contractor will contract for all institutions instead of one. This scheme was pressed by the department on the Clonmel Mental Hospital, Tipperary, but Dr. Harvey, the medical superintendent, said that he had sent a huge list to the department and received no reply. The committee, therefore, drafted a resolution to inform the Minister for Local Government, Dail Eireann, that his department sends no reply to its requests, that in consequence its private contracts have mostly expired, and that, in the circumstances, it has no alternative but to renew these contracts as soon as possible.

### A JOINT SCHEME FOR MANCHESTER.

THE joint scheme between the Manchester and Salford Saturday and Sunday Funds and the hospitals has now been drafted. Its object is to make the city's contribution to the voluntary hospitals compare less badly than it does now with the sums raised by similar means in other, and smaller, cities. The basis of the common action now determined is that no hospitals shall be deprived of existing collections for its benefit, but that in future no new collections are to be arranged by any hospital without the consent of the joint committee. This committee comprises sixteen members, eight of whom represent the hospitals. It will both manage the collections and distribute the product.

### PATIENTS AND THE HOSPITAL ATMOSPHERE.

WE have lately met a new criticism of the voluntary hospital which is worth noting, because it is evidently symptomatic of the gradual change, whereby paying patients of one kind or another are being admitted to the wards. In the interests of these it is asserted that various details bear the stamp, in the derogatory sense, of charity ; that the bread is served too thick, that the mugs are too coarse, and the like. We have often maintained that the weak point of hospital diet is not its quality, but its service ; that good food is often made unappetising through want of care in serving it. The objection to the early hour at which the patients are roused is a classic one ; yet we are always told that this is impossible to remedy. It certainly is so long as there is no will to remedy it ; but it can hardly be more impossible than the eight-hour day for nurses, which is now in effect the rule. It will be interesting to see what changes the paying patient brings to details like this, so long criticised but still taken for granted.

THE Chelsea Hospital for Women has received from its chairman, Sir Frederick Eley, a donation of £1,000.

THE first egg week held for the benefit of Essex County Hospital, Colchester, produced 17,000 eggs, the money value of which is over £100.

A DIAMOND tiara worth £300 or £400 has been presented by an anonymous lady to the Benn Ulster Eye, Ear and Throat Hospital, Belfast.

THE new wing at Merthyr General Hospital, given by Mr. Seymour Berry, is now complete. Its cost is estimated at £11,000.

AN Association of Domestic Workers has been formed in connection with the League of Mercy. The Central Committee of Domestic Workers formed last year has already raised a useful sum for the hospitals, and it is hoped to start local committees in such districts as Hampstead.

A survey of the financial condition of fifteen principal voluntary hospitals in Birmingham shows that during the past five years there has been a total deficiency of £210,404. Sir John Barnsley, well known in the hospital and business world of the city, has explained the position to a meeting of business men.

THE penny-in-the-pound scheme for the benefit of the hospitals in Sheffield has been adopted by 760 firms, representing 72,000 workers. In times of normal trade the scheme is expected to be supported by 150,000 contributors. The debt on the Sheffield hospitals is £120,000, and 400 more beds are needed there.

WORKMEN'S and employers' contributions amounted to 75 per cent. of the income of the Chesterfield Royal Hospital last year. The accounts show a surplus of £30, the most satisfactory balance-sheet ever presented. Mr. E. C. Barnes, the hospital's Chairman, believes this proportion to be a record for the whole country.

THE Dorset County Hospital has started an employees' insurance scheme, whereby any contributor of 1d. a week, or 2d. for a married man, after three months' contributions is entitled to admission at 9d. a day (instead of 1s. 6d.), and after six months' contributions to admission free. An immediate payment of 2s. 2d. is often adopted by new contributors.

THE League of the Roses, started at Islington in 1910, is an organisation of women and girls to raise funds for the maintenance of the women's and children's wards in the Royal Northern Hospital. For some years its activities were principally confined to Islington, each of the four Parliamentary divisions of the Borough being represented by its own committee, but additional branches have since been formed at Highgate and at Southgate (which includes Palmers Green and Winchmore Hill). Money is raised by means of collecting boxes, by sales of work, whist drives, dances, &c., and a number of donations and subscriptions. The League handed over £1,900 to the hospital last year.

THE women telegraphists of the Central Telegraph Office are trying to raise £3,000 to endow one bed at the Garrett Anderson Hospital for Women, the London Hospital and St. Bartholomew's.

MAJOR TRAFFORD has handed a sum of £117 to the Norfolk and Norwich Hospital, being the proceeds of the sale of the rifles bought by the public at the beginning of the war for the 1st Volunteer Battalion of the Norfolk Regiment.

IT is hoped to repeat this year the pit pony races at Doncaster for the benefit of the hospitals in the areas from which competitors come. Part of the St. Leger course will probably be granted. Last year £900 was so raised.

THE late Mr. William Ward, of Mellor, near Blackburn, has left the residue of his estate, estimated at £60,000, to the Blackburn and East Lancashire Royal Infirmary. The town has proposed to extend the infirmary at a cost of £100,000, towards which this bequest should be very useful.

THE late Mr. George Sheward, who died at the age of 90 and left estate valued at £355,000, has bequeathed the residue of his property, after certain bequests, equally to the London, Middlesex and St. Bartholomew's Hospitals. Though death duties absorb nearly one-third, the institutions should benefit considerably.

THE Nottingham General Hospital has had an unusually successful year, in which the receipts are £4,000 to the good, and the expenses £10,000 less on a turnover of £60,000. Despite the remaining overdraft of £10,000, Mr. Acton, the new president, states that no better report has ever been presented, and he has served the institution for nearly thirty years. The out-patient department, however, is in urgent need of improvement. The hospital is the residuary legatee of an estate valued at £48,000 net.

THE Earl of Donoughmore, at the annual meeting of the London Homœopathic Hospital, said that at the end of 1920 an accumulated deficiency of £27,700 was met by the withholding from investments of legacies and by a grant from the National Relief and King Edward's Fund of £9,800. The income from subscriptions in 1921 showed a decrease of £264. There was a gleam of hope in the fact that the income for 1921 had increased by £629 and the expenditure had decreased by £1,747.

SIR ERNEST HATCH, at the annual general meeting of the Corporation of University College Hospital, stated that owing to the receipt at the last moment of a grant of £12,000, the debit balance amounted to about £1,300, compared with £9,000 at the end of the previous year. The grant came at a very critical moment, when the Committee had been reduced to the last resort of obtaining authority to pledge the site of the hospital. The income, apart from the grant, amounted to £58,073, compared with £51,046 in 1920. The expenditure totalled £71,315, an increase of £630.

